# Osteomyelitis Generalis

**Published:** 1898-07

**Authors:** A. B. Campbell

**Affiliations:** V.S. (O. V. College), Berlin, Ontario


### OSTEOMYELITIS GENERALIS.

By A. B. Campbell, V.S. (O. V. College),

BERLIN, ONTARIO.

WAS called to see this horse in consultation with Dr. Orr, of Baden, who was undecided as to the nature of the case.

*Subject:* Driver, gelding, four years old.

*History:* For some months previous the animal showed great fatigue at moderate work, and in the stable assumed the recumbent position the greater part of the time. These symptoms continued to become more marked, and later, when standing, the back became arched, with gradual loss of condition. The horse had been treated by a local non-graduate veterinarian for kidney-trouble and inflammation of the spine.

Examination failed to reveal anything of importance in connection with any of the vital organs. Rectal examination failed to reveal any abnormal lesions, and a diagnosis by exclusion was aimed at, but the only conclusions arrived at on our first examination were the location of the trouble posteriorly, the right extremity affected more than the left, and only tonic treatment of a general character prescribed.

*Progress.* Some weeks later we found all the symptoms more marked, greater emaciation, lassitude, unable to retain the weight of the hind limbs but for a short time. The hind feet extended forward under the body, like a horse with laminitis, back more arched, and pulse and temperature very little disturbed.

There was one symptom which I could not understand until after the postmortem. The floor of the stall being cobblestone, there was one place where he would always search and feel for and would stand with the toes elevated on the edge of a stone, with the heels low in a hole. In this position the owner told us he seemed to get the most relief. From this time he was turned out to grass, with the request to the owner that he inform us if there was no improvement or the animal died. He continued to lose condition until he became unable to stand sufficient time to graze and was nothing but skin and bone, when we were given permission to destroy him.

*Autopsy.* All the internal organs were free from organic changes. The muscles of the femoral region were of a very pale whitish appearance, especially those attached to the femur. On sawing through the femur we noted the medullary portion had the appearance of clotted blood the entire length of the bone, and in the cancellated structure of the superior portion several points of pus-formation were apparent. The synovia of the pelvic and femoro-tibial articulations were slightly discolored with degenerative changes in the cartilage and coagulation of the synovial fluid, in part free or attached to the articular surfaces. The altered condition of the medullary portion of the bones was more marked and advanced in the right limb, and was noticeable in the bones of the fore limbs.

We pronounced the case osteomyelitis, but were unable to give any cause for it. No evidence of any traumatic origin or history of any injury. The peculiar position assumed by the animal while standing indicated that the weight of the parts was in this way supported more by the muscles than by the bony columns. This case corresponds in my mind very closely to what is termed in human medicine "hip-joint disease," and would in time in this case probably have terminated in a fistulous abscess.

---

A barking dog, a runaway, a lawsuit, a verdict for $5000 damages, the sale of Ex-President Hayes' home by the sheriff for the damages and costs amounting in all to $6095, are now a matter of record.

